# Direct Social Support and 19-Year Mortality Among Older Adults With Type 2 Diabetes

**DOI:** 10.1093/geroni/igaf122.1366

**Published:** 2025-12-31

**Authors:** Weidi Qin, Emily Nicklett, Deven Murray

**Affiliations:** University of Wisconsin–Madison, Madison, Wisconsin, United States; The University of Texas at San Antonio, San Antonio, Texas, United States; The University of Texas at San Antonio, San Antonio, Texas, United States

## Abstract

While existing research suggests that social support improves diabetes management and outcomes, the long-term impact on longevity remains unaddressed. To bridge this gap, the present study examines two questions: 1) Is direct social support associated with mortality among older adults with diabetes? 2) Is the interaction between social support and depressive symptoms associated with mortality? Older adults with type 2 diabetes were selected from the Health and Retirement Study’s 2003 Diabetes Study (n = 1,139). The year of death was ascertained by exit interview or spouse respondents through 2022. Direct social support was assessed with an 8-item scale indicating whether participants could count on their family or friends to help with diabetes management. Depressive symptoms were measured using CESD-8. Cox proportional hazards models were performed to test the study aims. From 2003 to 2020, 75.43% of participants died. Compared to low social support, high social support was associated with 1.58 times higher hazards of death (p = 0.016). The interaction between social support and depressive symptoms was significantly associated with mortality (hazard ratio = 2.39, p = 0.033). Specifically, among older adults with diabetes who had low social support, those with no depressive symptoms had lower hazards of mortality compared to those with mild depressive symptoms. The findings suggest that high social support is related to higher hazards of mortality among older adults with type 2 diabetes. It is important for diabetes management plans to incorporate strategies for leveraging social support to prolong the life expectancy among older adults.

